# Carnosic Acid Activates the STING/IRF3 Pathway to Induce Nitric Oxide-Mediated Apoptosis in Osteosarcoma Cells

**DOI:** 10.3390/antiox15030374

**Published:** 2026-03-16

**Authors:** Weixiong Guo, Lanlan Yin, Qiang Wu, Jiaqi Chu

**Affiliations:** 1 Orthopaedic Center, Affiliated Hospital of Guangdong Medical University, Zhanjiang 524001, China; 3230007501@student.must.edu.mo; 2 The State Key Laboratory of Quality Research in Chinese Medicine, Macau University of Science and Technology, Macau 999078, China; 3230005071@student.must.edu.mo; 3 Reproductive Medicine Center, Affiliated Hospital of Guangdong Medical University, Zhanjiang 524001, China; 4 Stem Cell Research and Cellular Therapy Center, Guangdong Provincial Key Laboratory of Autophagy and Major Chronic Non-Communicable Diseases, Affiliated Hospital of Guangdong Medical University, Zhanjiang 524001, China

**Keywords:** carnosic acid, osteosarcoma, apoptosis, STING/IRF3 pathway, nitric oxide

## Abstract

Osteosarcoma (OS) is a highly aggressive bone cancer with limited therapeutic options. Carnosic acid (CA), a phenolic diterpene with well-established antioxidant properties, has shown anticancer activity, yet its mechanisms in OS remain unclear. In this study, we found that CA suppressed proliferation and induced apoptosis in human osteosarcoma cells in a dose-dependent manner. Mechanistically, CA activated the STING/IRF3 signaling pathway and enhanced nitric oxide (NO) production, factors closely linked to redox modulation and mitochondrial apoptotic signaling. Pharmacological inhibition or siRNA-mediated knockdown of STING, as well as blockade of NO synthesis, significantly reduced CA-induced apoptosis in vitro. In a xenograft mouse model, CA treatment suppressed tumor growth, and this effect was partially reversed by STING inhibition. These findings suggest that CA exerts antitumor effects in OS through modulation of innate immune and redox-related signaling pathways, supporting its potential as a therapeutic compound that links antioxidant and immunomodulatory actions.

## 1. Introduction

Osteosarcoma (OS) is a highly aggressive primary bone malignancy that primarily affects children and adolescents [1]. Despite advances in surgical techniques and multi-agent chemotherapy, the prognosis for patients with metastatic or recurrent OS remains poor, with a five-year survival rate below 30% [2,3]. This highlights the urgent need for novel therapeutic strategies that not only inhibit tumor proliferation but also modulate the tumor microenvironment.

In recent years, increasing attention has been paid to the interplay between innate immune signaling and redox regulation in cancer therapy [4,5]. Immunotherapy has demonstrated promising outcomes in several solid tumors, but its efficacy in OS is limited due to immune evasion, low tumor antigenicity, and insufficient infiltration of cytotoxic T cells [6]. These challenges necessitate approaches that activate innate immune pathways while restoring immune-responsive conditions within the tumor microenvironment [7,8].

The cyclic GMP-AMP synthase (cGAS)–stimulator of interferon genes (STING) pathway is a key cytosolic DNA-sensing mechanism that triggers the phosphorylation of interferon regulatory factor 3 (IRF3) via TANK-binding kinase 1 (TBK1), leading to the production of type I interferons and other inflammatory mediators [9,10,11]. STING activation has been reported to reduce OS metastasis, enhance dendritic cell maturation, and improve CD8^+^ T cell function, highlighting its therapeutic potential [8,12].

Nitric oxide (NO), a small gaseous signaling molecule primarily generated by inducible nitric oxide synthase (iNOS), exerts diverse roles in tumor biology depending on its concentration and context. At high levels, NO contributes to oxidative stress, induces DNA damage, and promotes apoptosis, whereas at low or sustained levels, it may facilitate tumor progression, angiogenesis, and immune suppression [13,14,15,16]. Moreover, increasing evidence suggests a functional link between STING signaling and NO production, though the regulatory mechanisms are not fully understood [17]. Notably, NO serves as a redox mediator, and its interplay with innate immune activation represents a promising axis for anticancer interventions [15].

Carnosic acid (CA), a phenolic diterpene extracted from *Rosmarinus officinalis*, possesses well-documented antioxidant properties and broad antitumor activity against various cancers, including those of the prostate, lung, colon, and stomach [18]. Beyond its antioxidant potential, CA has been shown to interfere with cancer cell proliferation, migration, and mitochondrial function by modulating redox status and key signaling pathways such as PI3K/Akt, mTOR, and JAK/STAT3 [19,20,21,22,23]. More recently, CA has been implicated in immunomodulation and NO regulation, positioning it as a candidate molecule at the intersection of oxidative and immune signaling [9,24].

In this study, we investigated the pro-apoptotic effects of CA in osteosarcoma cells and delineated the involvement of the STING/IRF3 axis and NO signaling. Our findings suggest that CA mediates its antitumor effects through activation of innate immune and redox pathways. Although CA’s anticancer properties have been described in other malignancies, its specific actions in osteosarcoma remain insufficiently explored. A prior report demonstrated that CA selectively targets OS cells over healthy bone cells in vitro, but mechanistic and in vivo validation are lacking [25]. Our study aims to fill this gap and provide insights into the therapeutic potential of redox-active phytochemicals in osteosarcoma.

## 2. Materials and Methods

### 2.1. Reagents and Antibodies

Carnosic acid (CA, purity ≥ 98%) (MedChemExpress, Monmouth Junction, NJ, USA; Cat# HY-N0644R) was dissolved in dimethyl sulfoxide (DMSO) to obtain a 100 mM stock solution. The STING inhibitor H-151 (Cat# HY-112693) was also sourced from MCE. The nitric oxide assay kit based on the Griess reaction was provided by Beyotime Biotechnology (Shanghai, China). Dulbecco’s Modified Eagle’s Medium (DMEM), fetal bovine serum (FBS), penicillin–streptomycin solution, and trypsin–EDTA were obtained from Gibco (Thermo Fisher Scientific, Waltham, MA, USA).

Small interfering RNAs (siRNAs) specific for human STING (siSTING) and the negative control (siCTL) were synthesized by GenePharma (Shanghai, China). Transient transfections were performed using Lipofectamine™ 3000 reagent (Thermo Fisher Scientific, Waltham, MA, USA; Cat# L3000008) according to the manufacturer’s instructions.

For Western blotting and immunohistochemistry, primary antibodies included anti-STING (CST, Danvers, MA, USA; Cat# 13647), anti-TBK1 (CST, Cat# 3504), anti-p-TBK1 (Ser172, CST, Cat# 5483), anti-IRF-3 (CST, Cat# 11904), anti-p-IRF-3 (Ser396, CST, Cat# 4947), anti-cleaved caspase-3 (CST, Cat# 9664), anti-PARP (CST, Cat# 9542), anti-Bax (CST, Cat# 5023), anti-Bcl-2 (CST, Cat# 3498), anti-Bak (CST, Cat# 12105), anti-Bcl-xL (CST, Cat# 2764), and anti-α-Tubulin (CST, Cat# 2148). Alexa Fluor 488- or 594-conjugated secondary antibodies (Thermo Fisher Scientific, Waltham, MA, USA) were used for immunofluorescence. Enhanced chemiluminescence (ECL) detection reagents were purchased from Bio-Rad Laboratories (Hercules, CA, USA).

All chemicals were of analytical grade or higher, and deionized water was used throughout the experiments.

### 2.2. Cell Culture and Treatments

Human osteosarcoma cell lines HOS (catalog no. TCHu167) and MG63 (catalog no. TCHu124) were obtained from the Cell Bank of the Chinese Academy of Sciences (Shanghai, China). These cell lines represent osteoblast-like osteosarcoma models with distinct biological characteristics. MG63 cells are generally considered to exhibit an immature osteoblast-like phenotype with relatively high proliferative capacity, whereas osteosarcoma cell lines such as HOS represent heterogeneous osteoblastic phenotypes with different differentiation characteristics [26,27,28]. For the in vivo xenograft experiments, MG63 cells were selected because they are widely used in osteosarcoma tumorigenicity studies and exhibit stable tumor formation in nude mice. Cells were maintained in DMEM supplemented with 10% FBS, 100 U/mL penicillin, and 100 μg/mL streptomycin, under standard conditions of 37 °C in a humidified atmosphere containing 5% CO_2_.

Primary human bone marrow-derived mesenchymal stem cells (hBMSCs) were isolated from bone marrow aspirates collected during hip arthroplasty procedures performed on five female patients (aged 60–65 years) at the Affiliated Hospital of Guangdong Medical University. Written informed consent was obtained from all donors. The protocol adhered to the Declaration of Helsinki and received approval from the Medical Ethical Committee of the Affiliated Hospital of Guangdong Medical University (Approval No. GXB2024-001-02). Mononuclear cells were separated by density gradient centrifugation with Histopaque-1077 (Sigma-Aldrich, St. Louis, MO, USA). The buffy coat was seeded into culture flasks containing α-MEM (Gibco, Thermo Fisher Scientific, Waltham, MA, USA) supplemented with 10% (*v/v*) FBS, 10 U/mL penicillin G, and 10 μg/mL streptomycin, and maintained at 37 °C in a 5% CO_2_ incubator. Medium was refreshed every three days. When cell confluence reached approximately 90%, cultures were digested with 0.25% trypsin–EDTA and subcultured at a 1:3 ratio. Cells from passages 4–7 were used in subsequent assays.

For in vitro experiments, CA stock solution (prepared in DMSO) was diluted in culture medium to final concentrations of 5, 10, 20, or 40 μM. The final DMSO concentration did not exceed 0.1%. Control cells received the same volume of vehicle. For STING pathway inhibition, cells were pre-incubated with the selective STING inhibitor H-151 (10 μM) for 1 h prior to CA treatment. Gene knockdown was performed in HOS and MG63 cells using 100 nM siRNA targeting STING (siSTING) or scrambled control siRNA (siCTL) (GenePharma, Shanghai, China) delivered with Lipofectamine™ 3000 reagent (Thermo Fisher Scientific, USA) following the manufacturer’s protocol. Knockdown efficiency was confirmed by Western blotting 48 h after transfection.

In experiments assessing NO signaling, cells were pretreated with the non-selective NOS inhibitor NG-monomethyl-L-arginine (L-NMMA; Sigma-Aldrich, St. Louis, MO, USA) for 4 h before CA exposure. The working concentration of L-NMMA was determined based on prior literature and preliminary optimization. All assays were performed in triplicate and independently repeated at least three times to ensure reproducibility.

### 2.3. Cell Viability and Proliferation Assays

Cell viability was determined using the CellTiter 96^®^ AQueous One Solution Cell Proliferation Assay kit (MTS assay; Promega, Madison, WI, USA) following the manufacturer’s protocol. HOS and MG63 cells were seeded into 96-well plates (5 × 10^3^ cells/well) and allowed to adhere overnight before treatment with CA at final concentrations of 0~320 μM for 12, 24, or 36 h. At each time point, 20 μL of MTS reagent was added to each well, followed by incubation at 37 °C for 2 h. Absorbance was read at 490 nm using a microplate reader (BioTek Instruments, Winooski, VT, USA). Data were expressed as the percentage of viable cells relative to untreated controls.

Colony formation ability was evaluated to assess long-term proliferative potential. HOS and MG63 cells were seeded in 6-well plates (500 cells/well) and allowed to attach for 24 h prior to treatment with CA (0~40 μM) or vehicle control (0.1% DMSO). Cultures were maintained for 10–14 days, with medium replaced every three days. Colonies were then fixed in 4% paraformaldehyde (15 min) and stained with 0.1% crystal violet (30 min).

### 2.4. Wound Healing and Migration Assay

The influence of CA on osteosarcoma cell migration was assessed using a scratch wound assay. HOS and MG63 cells were seeded into 6-well plates and cultured until a confluent monolayer (90–100%) was formed. A sterile 200 μL pipette tip was then used to generate a straight scratch across the monolayer. Detached cells were removed by washing twice with phosphate-buffered saline (PBS), and cultures were maintained in serum-free DMEM containing CA (0~40 μM). Images of the wound area were captured at 0 h and 24 h using a phase-contrast microscope (Olympus Corporation, Tokyo, Japan) for subsequent analysis.

### 2.5. Flow Cytometry for Apoptosis

Apoptosis induction by CA was quantified via Annexin V-FITC/propidium iodide (PI) staining followed by flow cytometric analysis. HOS and MG63 cells were seeded into 6-well plates and exposed to CA (40 μM) for 24 h. Where indicated, cells were pre-incubated with the STING inhibitor H-151 (10 μM) for 1 h before CA treatment. After exposure, both adherent and floating cells were collected, washed twice with cold PBS, and resuspended in 1× binding buffer at a final density of 1 × 10^6^ cells/mL. Cell suspensions were stained with 5 μL Annexin V-FITC and 5 μL PI (Annexin V-FITC/PI Apoptosis Detection Kit; Beyotime Biotechnology, Shanghai, China) for 15 min in the dark at room temperature. Stained samples were analyzed within 1 h using a BD FACSCanto™ II flow cytometer (BD Biosciences, San Jose, CA, USA). Data were processed with FlowJo software (version 10.8, BD Biosciences, Ashland, OR, USA), and the percentages of early (Annexin V^+^/PI^−^) and late (Annexin V^+^/PI^+^) apoptotic cells were determined. The sum of both populations represented the total apoptotic fraction. All assays were independently repeated at least three times, and results were reported as mean ± SD.

### 2.6. NO Detection and NOS Expression

NO levels in osteosarcoma cell cultures were quantified using a Griess Reagent Kit (Beyotime Biotechnology, Shanghai, China) in accordance with the manufacturer’s protocol. HOS and MG63 cells were seeded into 6-well plates and treated with CA at final concentrations of 0, 10, or 20 μM for 24 h. In designated groups, cells were pre-incubated with the STING inhibitor H-151 (10 μM) for 1 h prior to CA exposure or transfected with STING-targeting siRNA (siSTING) 48 h before CA treatment. For NO determination, cells were washed and subsequently incubated in phenol red–free DMEM containing low serum (1% FBS) during the NO measurement period to minimize background interference from serum components. Following treatment, supernatants were collected and centrifuged at 1000× *g* for 10 min at room temperature to eliminate debris. Equal volumes (50 μL) of the clarified supernatant and Griess reagent were combined in a 96-well plate and incubated for 10 min at room temperature in the dark. Absorbance was read at 540 nm using a microplate reader (BioTek Instruments, Winooski, VT, USA). A sodium nitrite standard curve was generated, and NO concentrations in samples were calculated accordingly.

Total RNA was isolated from cells using TRIzol reagent (Invitrogen, Thermo Fisher Scientific, Waltham, MA, USA) and reverse-transcribed with the PrimeScript RT Reagent Kit (Takara Bio Inc., Kusatsu, Japan). Quantitative PCR was performed using TB Green Premix Ex Taq II (Takara Bio Inc., Kusatsu, Japan) on a CFX96 Real-Time PCR Detection System (Bio-Rad Laboratories, Hercules, CA, USA). Expression of NOS isoforms was normalized to GAPDH, and relative changes were calculated by the 2^−ΔΔCt^ method. The primers used were as follows: nNOS forward: 5′-ACA CGC ATG TCT GGA AAG GCA C-3′; nNOS reverse: 5′-CTC TGT GGC ATA GAG GAT GGT C-3′; eNOS forward: 5′-GAA GGC GAC AAT CCT GTA TGG C-3′; eNOS reverse: 5′-TGT TCG AGG GAC ACC ACG TCA T-3′; iNOS forward: 5′-GCT CTA CAC CTC CAA TGT GAC C-3′; iNOS reverse: 5′-CTG CCG AGA TTT GAG CCT CAT G-3′; GAPDH forward: 5′-GAA GGT GAA GGT CGG AGT C-3′; GAPDH reverse: 5′-GAA GAT GGT GAT GGG ATT TC-3′. All qPCR reactions were performed in triplicate. Relative mRNA expression levels were reported as mean ± SD and analyzed using one-way ANOVA followed by Dunnett’s post hoc test.

### 2.7. Western Blot Analysis

Western blotting was performed to evaluate the expression of apoptosis-related proteins and STING signaling molecules in osteosarcoma cells. Following the indicated treatments with CA, H-151, or siSTING, HOS and MG63 cells were lysed in RIPA buffer (Beyotime, China) supplemented with protease and phosphatase inhibitors. Protein concentrations were determined using a BCA assay kit (Beyotime, China). Equal amounts of protein (30–40 μg) were resolved on 10–12% SDS-PAGE gels and transferred onto polyvinylidene fluoride (PVDF) membranes (Millipore, Billerica, MA, USA). Membranes were blocked in 5% non-fat milk prepared in Tris-buffered saline with 0.1% Tween-20 (TBST) for 1 h at room temperature, followed by overnight incubation at 4 °C with primary antibodies against STING, TBK1, p-TBK1 (Ser172), IRF-3, p-IRF-3 (Ser396), cleaved caspase-3, PARP, Bax, Bcl-2, and α-Tubulin as loading control. After TBST washes, membranes were incubated with horseradish peroxidase (HRP)-conjugated secondary antibodies (1:5000) for 1 h at room temperature. Protein signals were detected using an ECL ultra-sensitive chemiluminescent substrate (Merck Millipore, Darmstadt, Germany; Cat# WBKLS0100) and visualized with a ChemiDoc MP imaging system (Bio-Rad, USA). Band intensities were quantified using ImageJ software (version 1.53, National Institutes of Health, Bethesda, MD, USA). A consistent region of interest (ROI) was applied to each band, and background signal was subtracted before densitometric measurement. Protein expression levels were normalized to α-Tubulin. For cytoplasmic and nuclear protein fractionation, fibrillarin and α-Tubulin were used as nuclear and cytoplasmic markers, respectively, to confirm fraction purity. For phosphorylated proteins, band intensities were additionally normalized to their respective total protein levels where indicated. Relative protein expression was calculated by setting the control group to 1.0. All Western blot experiments were independently repeated at least three times. Quantitative data are presented as mean ± SD. Statistical comparisons were performed using one-way ANOVA followed by Dunnett’s post hoc test, with the control group serving as the reference.

### 2.8. Immunofluorescence Staining

Immunofluorescence staining was used to determine the subcellular distribution of STING and phosphorylated IRF-3 (p-IRF-3) in osteosarcoma cells after CA exposure. HOS and MG63 cells were seeded on sterile glass coverslips placed in 24-well plates and treated with CA (10 or 20 μM) for 24 h, with or without prior incubation with the STING inhibitor H-151 (10 μM). Following treatment, cells were fixed in 4% paraformaldehyde for 15 min at room temperature, permeabilized with 0.1% Triton X-100 for 10 min, and blocked with 5% bovine serum albumin (BSA) for 1 h. Primary antibody incubation was performed overnight at 4 °C using anti-STING (1:200) or anti–p-IRF-3 (1:200). After PBS washes, cells were incubated with Alexa Fluor 488– or 594–conjugated secondary antibodies (1:500, Thermo Fisher Scientific, USA) for 1 h at room temperature in the dark. Nuclei were stained with DAPI (1 μg/mL) for 5 min. Coverslips were mounted in antifade mounting medium and examined using a Nikon A1 confocal laser scanning microscope (Nikon Corporation, Tokyo, Japan). All images were acquired under identical exposure conditions. ImageJ software was used for fluorescence intensity quantification and analysis of nuclear localization. Representative images from at least three independent experiments were evaluated.

### 2.9. siRNA Transfection

Small interfering RNAs (siRNAs) targeting human STING were designed and synthesized by GenePharma (Shanghai, China) using a “3 + 1 guarantee” strategy, in which three primary candidate sequences and one additional backup sequence were provided to ensure effective gene silencing. Four independent siRNA oligonucleotides were generated, designated as STING-homo-679, STING-homo-806, STING-homo-886, and STING-homo-1047. The siRNA sequences were as follows: STING-homo-679: Sense: 5′- GCC CUU CAC UUG GAU GCU UTT-3′, Antisense: 5′-AAG CAU CCA AGU GAA GGG CTT-3′; STING-homo-806: Sense: 5′-GGG CUG GCA UGG UCA UAU UTT-3′, Antisense: 5′-AAU AUG ACC AUG CCA GCC CTT-3′; STING-homo-886: Sense: 5′-GCA UUA CAA CAA CCU GCU ATT-3′, Antisense: 5′-UAG CAG GUU GUU GUA AUG CTT-3′; STING-homo-1047: Sense: 5′-GGG UUU ACA GCA ACA GCA UTT-3′, Antisense:5′-AUG CUG UUG CUG UAA ACC CTT-3′. Each siRNA candidate was individually transfected into HOS and MG63 cells, and knockdown efficiency was evaluated by Western blot analysis 48 h post-transfection. Among the four tested sequences, STING-homo-1047 consistently exhibited the highest knockdown efficiency and was therefore selected for all subsequent mechanistic and functional experiments. For transfection, HOS and MG63 cells were seeded into 6-well plates and transfected at ~60–70% confluence using Lipofectamine™ 3000 reagent (Thermo Fisher Scientific, USA) in accordance with the manufacturer’s protocol. For each well, 100 nM siRNA was diluted in Opti-MEM™ and combined with Lipofectamine™ 3000 for 15 min before being added to the cultures. After 6 h of incubation, the transfection medium was replaced with fresh complete medium, and cells were maintained for an additional 42–48 h prior to downstream experiments. Knockdown efficiency was verified by Western blot analysis. Following transfection, cells were subjected to NO quantification, apoptosis assays, and Western blotting to evaluate downstream signaling changes and functional outcomes. All transfection procedures were independently performed at least three times.

### 2.10. In Vivo Xenograft Tumor Model

A human osteosarcoma xenograft model was employed to assess the antitumor activity of CA in vivo. All procedures were reviewed and approved by the Ethical Review Committee of Guangdong Medical University (Approval No. AHGDMU-LAC-A-202403-013) and were conducted in accordance with national and institutional guidelines for the care and use of laboratory animals. Male BALB/c nude mice (6 weeks old, 18–22 g) were obtained from Changsheng Bio-Technology Inc. (Benxi City, Liaoning, China) and maintained under specific pathogen-free (SPF) conditions at 22 ± 2 °C, 55 ± 5% relative humidity, and a 12 h light/dark cycle, with ad libitum access to standard chow and water. MG63 cells were selected for the xenograft model because this osteosarcoma cell line is widely used in tumorigenicity studies and is known to exhibit reliable and stable tumor formation in nude mice [29,30]. MG63 cells (5 × 10^6^) suspended in 100 μL PBS were injected subcutaneously into the right flank. When tumor volumes reached ~100 mm^3^, animals were randomly assigned to four groups (*n* = 5 per group): vehicle control (PBS), CA (30 mg/kg every 2 days, intraperitoneal injection), H-151 (10 mg/kg every 2 days, intraperitoneal injection), and CA + H-151 combination. Treatments continued for 14 consecutive days. At study termination, mice were euthanized, and tumors were excised, photographed, and processed for histological examination (fixed in 4% paraformaldehyde) or snap-frozen in liquid nitrogen for protein extraction. Tumor volumes were calculated using the formula: volume = (length × width^2^)/2. The effects of CA and H-151 on tumor growth were evaluated based on tumor volume and molecular analysis of tumor tissue (IHC and Western blotting).

### 2.11. Histological and Immunohistochemical Analysis

Tumor specimens were fixed in 4% paraformaldehyde for 24 h, dehydrated, paraffin-embedded, and sectioned at 4 μm thickness. For histopathological examination, hematoxylin and eosin (H&E) staining was performed, and slides were observed under a light microscope (Nikon Corporation, Tokyo, Japan). For immunohistochemical (IHC) staining, paraffin sections were deparaffinized with xylene and rehydrated through graded ethanol solutions. Antigen retrieval was achieved by heating the sections in 10 mM sodium citrate buffer (pH 6.0) using a microwave for 15 min, followed by cooling to room temperature. Endogenous peroxidase activity was blocked with 3% hydrogen peroxide for 10 min, and nonspecific binding was reduced by incubation with 5% bovine serum albumin (BSA) for 1 h. Sections were then incubated overnight at 4 °C with primary antibodies targeting Ki67 (1:200, CST, Cat# 9027), STING (1:200, CST, Cat# 13647), p-TBK1 (1:200, CST, Cat# 5483), and p-IRF-3 (1:200, CST, Cat# 4947). The following day, slides were incubated with HRP-conjugated secondary antibodies for 1 h and developed using DAB (3,3′-diaminobenzidine) substrate. Nuclei were counterstained with hematoxylin. Images were captured using a Nikon DS-Fi3 digital microscope (Nikon Corporation, Tokyo, Japan), and five random high-power fields (×400) per section were analyzed.

### 2.12. Statistical Analysis

All quantitative data are presented as mean ± standard deviation (SD) from at least three independent biological experiments unless otherwise stated. Statistical analyses were performed using GraphPad Prism software (version 9.0, San Diego, CA, USA). Comparisons between two groups were conducted using an unpaired two-tailed Student’s *t* test. For comparisons among multiple groups, one-way or two-way analysis of variance (ANOVA) was applied as appropriate. When multiple treatment groups were compared with a single control group, Dunnett’s post hoc test was used. Tukey’s post hoc test was applied when all pairwise comparisons among groups were required. Tumor growth curves were analyzed using two-way ANOVA with repeated measures. For flow cytometry and Western blot densitometric analyses, quantitative values represent independent experimental replicates. For immunohistochemical analysis, staining was quantified where indicated, and analyses were performed in a blinded manner. A *p* value < 0.05 was considered statistically significant.

## 3. Results

### 3.1. CA Inhibits Proliferation and Migration of Osteosarcoma Cells In Vitro

To ensure that the observed effects were not limited to a single osteosarcoma cell model, experiments were performed in two osteosarcoma cell lines (HOS and MG63), which represent distinct osteoblastic phenotypes. As shown in the following experiments, CA treatment produced consistent effects in both cell lines.

**Figure 1:**
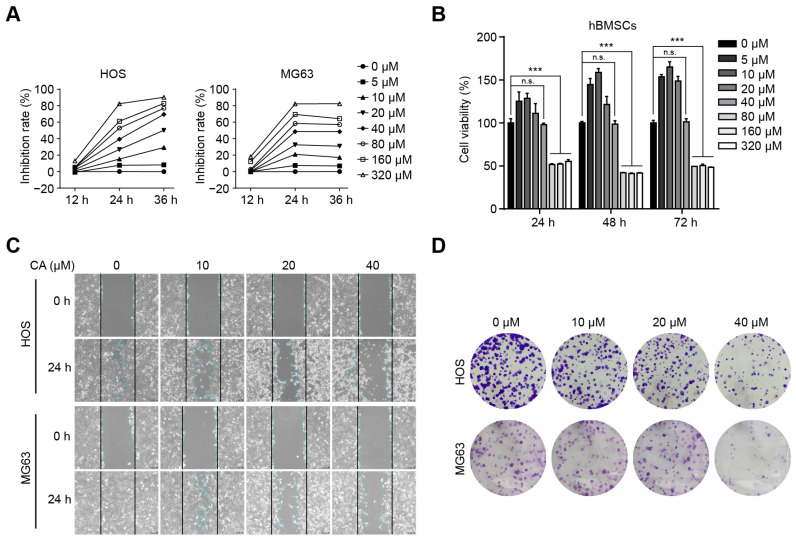
CA suppresses osteosarcoma cell proliferation and migration. (**A**) MTS assay results showing concentration-dependent inhibition of cell proliferation in HOS and MG63 cells after treatment with CA (0–320 μM) for 12, 24, and 36 h. Data are presented as mean ± SD from three independent biological experiments (*n* = 3). (**B**) Effect of CA on viability of normal hBMSCs, demonstrating minimal cytotoxicity below 40 μM. Data are presented as mean ± SD (*n* = 3). One-way ANOVA followed by Dunnett’s post hoc test (vs. control). n.s., no significant difference; *** *p* < 0.001. (**C**) Representative images from wound healing assays illustrating reduced migration capacity after CA treatment for 24 h. Black solid lines represent initial wound edges; blue dotted lines indicate cell migration boundaries. Images are representative of three independent experiments. (**D**) Colony formation assays showing dose-dependent inhibition of colony number and size in osteosarcoma cells treated with CA. Images are representative of three independent experiments.

To assess the growth-inhibitory potential of CA, HOS and MG63 cells were exposed to a gradient of concentrations (0–320 μM) for 12, 24, and 36 h, and cell viability was quantified using the MTS assay. CA reduced the proliferation of both osteosarcoma cell lines in a time- and dose-dependent fashion (Figure 1A). Importantly, the compound displayed selective cytotoxicity: concentrations ≤40 μM produced no significant effect on the viability of hBMSCs, whereas higher doses (≥80 μM) markedly decreased their survival (Figure 1B). Based on these findings, concentrations of 40 μM or lower were selected for subsequent assays to minimize off-target cytotoxicity.

A wound healing assay demonstrated that CA at 10–40 μM significantly impeded the migration of HOS and MG63 cells after 24 h (Figure 1C). Consistently, colony formation capacity was diminished in a dose-dependent manner, with both the number and size of colonies reduced (Figure 1D). These results indicate that CA selectively inhibits the proliferative and migratory abilities of osteosarcoma cells while sparing hBMSCs at lower concentrations.

### 3.2. CA Induces Apoptosis in Osteosarcoma Cells

**Figure 2:**
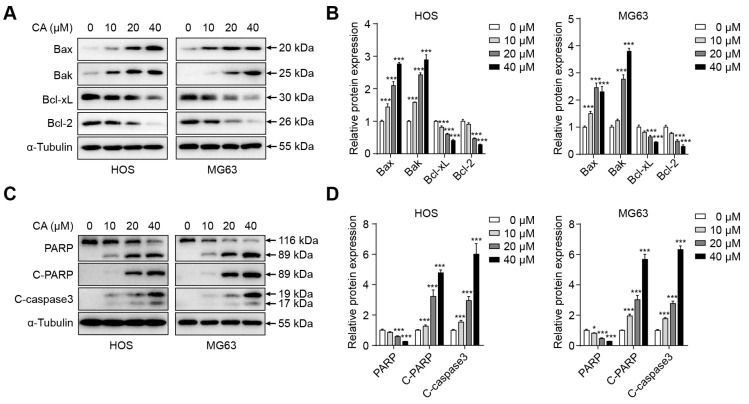
CA induces mitochondrial apoptosis in osteosarcoma cells. (**A**) Western blot analysis of mitochondrial apoptosis-related proteins in HOS and MG63 cells treated with CA (0, 10, 20, 40 μM) for 12 h. (**B**) Densitometric quantification of the proteins shown in (**A**) in HOS and MG63 cells. Band intensities were quantified using ImageJ, normalized to α-Tubulin, and expressed relative to the control group. (**C**) Western blot analysis of cleaved caspase-3 and cleaved PARP in HOS and MG63 cells after CA treatment. (**D**) Densitometric quantification of cleaved caspase-3 and cleaved PARP shown in (**C**) in HOS and MG63 cells. Band intensities were quantified using ImageJ, normalized to α-Tubulin, and expressed relative to the control group. Representative blots from three independent experiments are shown. Data are presented as mean ± SD (*n* = 3). Statistical significance was determined by one-way ANOVA followed by Dunnett’s post hoc test versus the control group. * *p* < 0.05, *** *p* < 0.001.

To determine whether CA promotes apoptosis in osteosarcoma cells, HOS and MG63 cells were exposed to graded concentrations of CA (0, 10, 20, and 40 μM) for 12 h. Western blot analysis revealed that Bax and Bak levels progressively increased with higher CA doses, whereas Bcl-2 and Bcl-xL levels decreased correspondingly in both cell lines (Figure 2A). Densitometric analysis further confirmed these dose-dependent changes (Figure 2B). Moreover, the expression of cleaved caspase-3 and cleaved PARP, two hallmarks of apoptosis, was markedly elevated following CA treatment (Figure 2C). Quantitative analysis demonstrated a significant increase in cleaved caspase-3 and cleaved PARP levels in response to CA exposure in both HOS and MG63 cells (Figure 2D). These findings suggest that CA induces apoptosis in osteosarcoma cells, likely through activation of the mitochondrial pathway by modulating the balance between pro- and anti-apoptotic proteins and promoting caspase-dependent cleavage of PARP.

### 3.3. CA Enhances NO Production by Upregulating NOS Expression

**Figure 3:**
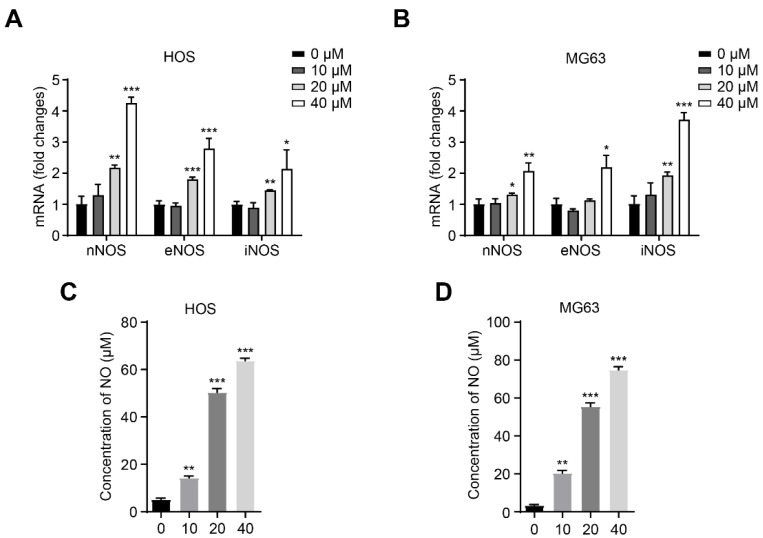
CA upregulates NOS isoforms and enhances NO production in osteosarcoma cells. (**A**,**B**) qRT-PCR results showing concentration-dependent upregulation of nNOS, eNOS, and iNOS mRNA levels in HOS and MG63 cells treated with CA (10, 20, and 40 μM) for 24 h. (**C**,**D**) Griess assay quantifying NO production in the culture medium after CA treatment, showing a significant increase in NO levels with increasing CA concentrations. Data are expressed as mean ± SD (*n* = 3). One-way ANOVA followed by Dunnett’s post hoc test (vs. control). * *p* < 0.05, ** *p* < 0.01, *** *p* < 0.001.

To determine whether CA affects NO metabolism in osteosarcoma cells, we analyzed the expression of nitric oxide synthase (NOS) isoforms after CA treatment. HOS and MG63 cells were exposed to CA (10, 20, and 40 μM) for 24 h, and the mRNA levels of nNOS, eNOS, and iNOS were measured by quantitative real-time PCR. The results showed that CA significantly upregulated these three NOS isoforms in a dose-dependent manner (Figure 3A,B). To assess whether these transcriptional changes led to functional NO production, NO levels in the culture medium were quantified using the Griess assay. As shown in Figure 3C,D, NO levels were significantly elevated in both HOS and MG63 cells following CA treatment, in a manner consistent with the increased expression of NOS genes. These findings indicate that CA promotes NO production in osteosarcoma cells by transcriptionally activating NOS isoforms.

### 3.4. NOS Inhibition Attenuates CA-Induced NO Production and Apoptosis in Osteosarcoma Cells

**Figure 4:**
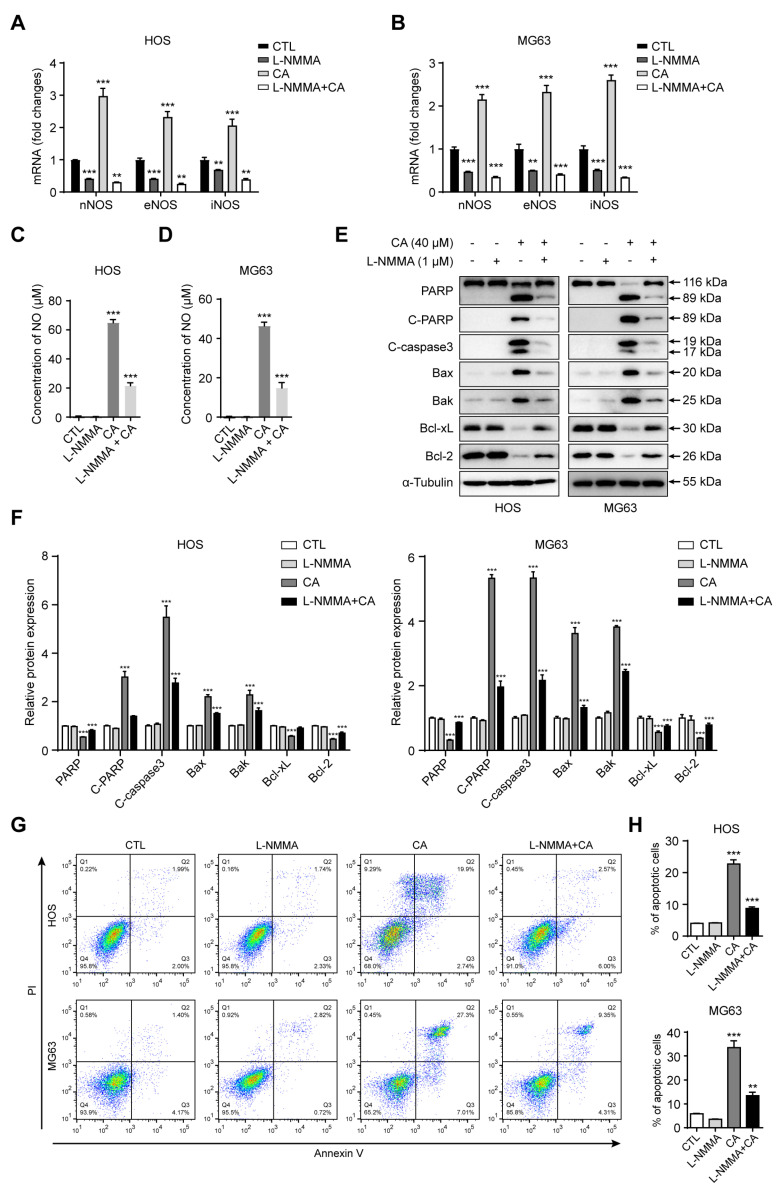
Inhibition of NOS reverses CA-induced NO production and apoptosis in osteosarcoma cells. (**A**,**B**) Relative mRNA expression levels of nNOS, eNOS, and iNOS in HOS and MG63 cells measured by qRT-PCR. Pretreatment with the NOS inhibitor L-NMMA (1 mM) markedly attenuated CA (40 μM)-induced upregulation of NOS transcription. (**C**,**D**) NO levels detected by Griess assay. L-NMMA significantly suppressed CA-induced NO production. (**E**) Western blot analysis of apoptosis-related proteins in HOS and MG63 cells. L-NMMA pretreatment reversed CA-induced upregulation of cleaved PARP, cleaved caspase-3, Bax, and Bak, and restored the expression of anti-apoptotic proteins Bcl-2 and Bcl-xL. (**F**) Densitometric quantification of the proteins shown in (**E**). Band intensities were quantified using ImageJ. (**G**) Flow cytometric analysis of apoptosis in HOS and MG63 cells. L-NMMA pretreatment reduced CA-induced apoptotic cell death compared with CA treatment alone. (**H**) Quantitative analysis of total apoptotic cells (early + late apoptosis) in HOS and MG63 cells. All data are presented as mean ± SD (*n* = 3). Statistical significance was determined by one-way ANOVA followed by Dunnett’s post hoc test. ** *p* < 0.01, *** *p* < 0.001 versus control.

To determine whether NO generation is a critical mediator of CA’s pro-apoptotic activity, we applied the broad-spectrum NOS inhibitor L-NMMA to suppress NO synthesis. HOS and MG63 cells were first incubated with 1 mM L-NMMA for 4 h and subsequently exposed to 40 μM CA for 24 h. qRT-PCR analysis showed that, compared with CA treatment alone, L-NMMA markedly decreased the mRNA expression of nNOS, eNOS, and iNOS (Figure 4A,B). Consistently, Griess assay results revealed a substantial reduction in extracellular NO levels following NOS inhibition (Figure 4C,D). We next examined whether NO suppression influences apoptosis. Western blot analysis demonstrated that L-NMMA pretreatment counteracted CA-induced increases in cleaved PARP, cleaved caspase-3, and the pro-apoptotic factors Bax and Bak, while restoring the expression of the anti-apoptotic proteins Bcl-2 and Bcl-xL (Figure 4E). Densitometric quantification further confirmed these changes (Figure 4F). In agreement with these findings, flow cytometric analysis showed that the proportion of total apoptotic cells (early + late apoptosis) was significantly reduced in the L-NMMA + CA group compared with cells treated with CA alone in both HOS and MG63 cells (Figure 4G,H). Taken together, these results suggest that NO production is indispensable for CA-driven mitochondrial apoptosis in osteosarcoma cells, and that pharmacological NOS inhibition effectively diminishes CA’s antitumor efficacy.

### 3.5. CA Activates the STING/IRF-3 Signaling Pathway and Promotes IRF-3 Nuclear Translocation

**Figure 5:**
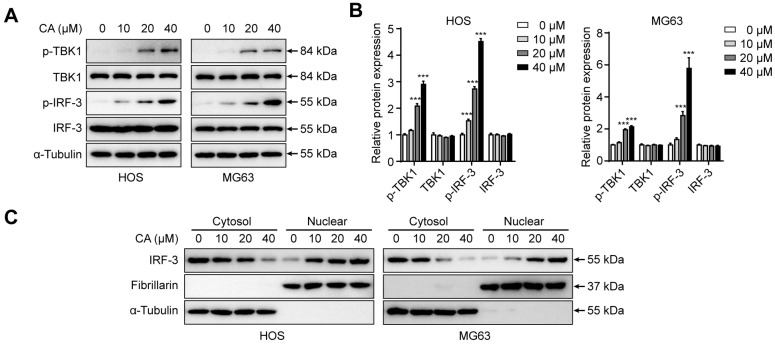
CA activates the STING/IRF-3 signaling pathway and induces IRF-3 nuclear translocation. (**A**) Western blot analysis of p-TBK1 and p-IRF-3 expression in HOS and MG63 cells treated with CA for 24 h at indicated concentrations (0–40 μM). (**B**) Densitometric quantification of p-TBK1 and p-IRF-3 protein levels shown in (**A**). Band intensities were quantified using ImageJ, normalized to α-Tubulin. (**C**) Cytoplasmic and nuclear fractions were analyzed for IRF-3 distribution. Fibrillarin and α-Tubulin were used as nuclear and cytoplasmic markers, respectively, to verify fraction purity. Quantification and statistical analysis apply to panels (**A**,**B**). Data in panel (**C**) are representative of three independent experiments. All data are presented as mean ± SD (*n* = 3). Statistical significance was determined by one-way ANOVA followed by Dunnett’s post hoc test. *** *p* < 0.001 versus control.

To elucidate the molecular mechanisms underlying CA-induced apoptosis, we investigated the activation of the STING pathway in osteosarcoma cells. HOS and MG63 cells were treated with increasing concentrations of CA (0, 10, 20, 40 μM) for 24 h, and the expression of STING pathway-related proteins was examined by Western blotting. As shown in Figure 5A, CA treatment led to a significant, dose-dependent increase in the phosphorylation levels of TBK1 (p-TBK1) and IRF-3 (p-IRF-3), while the total protein levels of TBK1 and IRF-3 remained relatively unchanged. Densitometric analysis further confirmed the significant upregulation of p-TBK1 and p-IRF-3 in response to CA exposure (Figure 5B). These results indicate that CA activates the STING signaling cascade in osteosarcoma cells. To further assess whether activated IRF-3 translocates into the nucleus, we separately extracted cytoplasmic and nuclear proteins and analyzed IRF-3 distribution. As shown in Figure 5C, CA treatment resulted in a gradual decrease in cytoplasmic IRF-3 and a corresponding increase in nuclear IRF-3 expression, indicating effective nuclear translocation of IRF-3 upon CA stimulation. Collectively, these results indicate that CA activates the STING/TBK1/IRF-3 signaling axis and promotes IRF-3 nuclear translocation, suggesting that this pathway may contribute to CA-induced apoptosis in osteosarcoma cells.

### 3.6. STING Knockdown Reverses CA-Induced IRF-3 Activation, NOS Expression, NO Production, and Apoptosis

**Figure 6:**
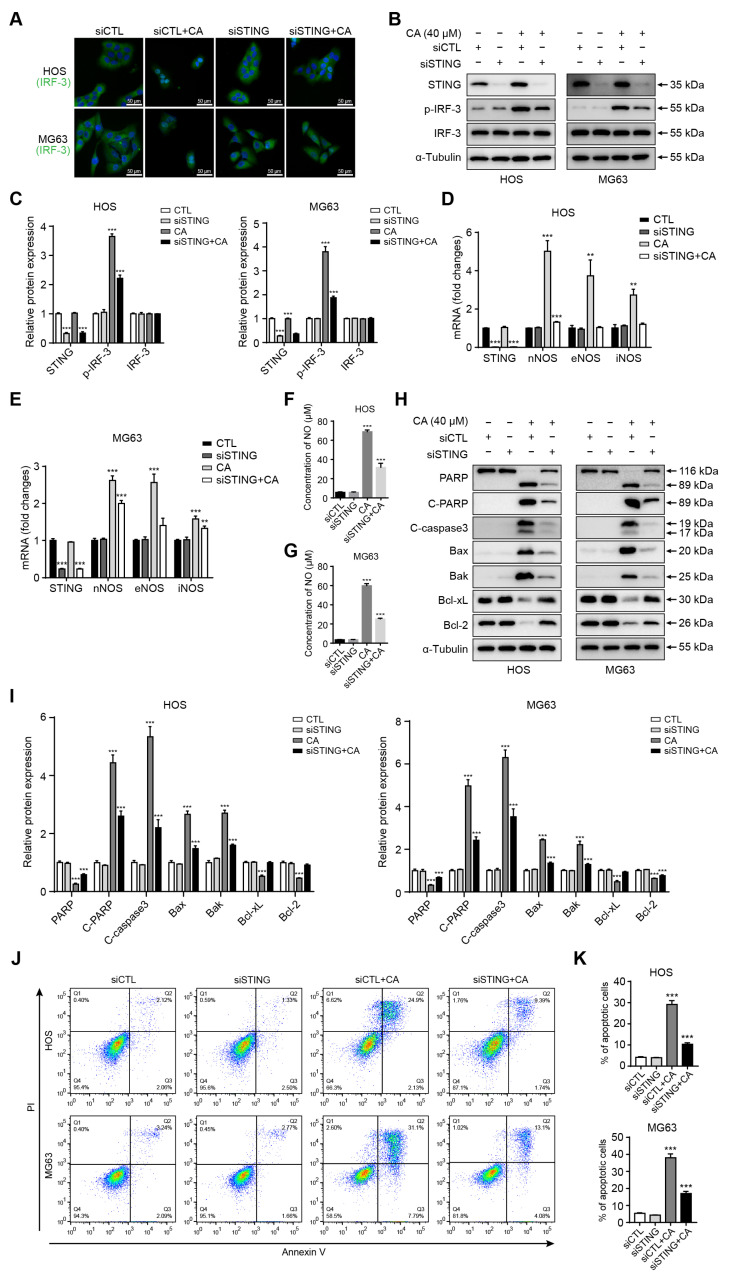
STING knockdown abrogates CA-induced STING/IRF-3 activation, NO production, and apoptosis. (**A**) Immunofluorescence staining showing IRF-3 nuclear translocation (green) and DAPI nuclear counterstain (blue). CA treatment (siCTL + CA) enhanced nuclear IRF-3, which was reduced upon STING knockdown (siSTING + CA). Scale bar = 50 μm. (**B**) Western blot analysis of p-IRF-3 expression in control and STING-silenced cells following CA treatment. (**C**) Densitometric quantification of p-IRF-3 protein levels shown in (**B**). Band intensities were quantified using ImageJ, normalized to α-Tubulin, and expressed relative to the control group. (**D**,**E**) qRT-PCR analysis of nNOS, eNOS, and iNOS expression in HOS and MG63 cells. CA-induced transcriptional upregulation was reversed by siSTING. (**F**,**G**) NO levels detected by Griess assay. STING knockdown significantly decreased NO production induced by CA. (**H**) Western blot analysis of apoptosis-related proteins (cleaved PARP, cleaved caspase-3, Bax, Bak, Bcl-2, and Bcl-xL) in control and STING-silenced cells after CA treatment. (**I**) Densitometric quantification of the proteins shown in (**H**). Band intensities were quantified using ImageJ, normalized to α-Tubulin, and expressed relative to the control group. (**J**) Flow cytometry analysis of apoptosis in HOS and MG63 cells. STING knockdown reduced CA-induced apoptotic cell death. (**K**) Quantitative analysis of total apoptotic cells (early + late apoptosis) in HOS and MG63 cells. Data are presented as mean ± SD (*n* = 3). Statistical significance was determined by one-way ANOVA followed by Dunnett’s post hoc test. ** *p* < 0.01, *** *p* < 0.001 versus control.

To ensure the specificity and efficiency of STING knockdown, four independent siRNAs targeting different regions of the STING mRNA were initially screened. Western blot analysis demonstrated that STING-homo-1047 achieved the highest knockdown efficiency in both HOS and MG63 cells and was therefore selected for subsequent experiments (Figure S1). To determine whether CA exerts its pro-apoptotic effects via the STING/IRF-3 pathway, STING expression was silenced by siRNA in HOS and MG63 cells prior to CA (40 μM) treatment. Immunofluorescence staining demonstrated that CA treatment (siCTL + CA group) markedly promoted IRF-3 nuclear translocation, whereas this effect was significantly attenuated in the siSTING + CA group (Figure 6A). Consistently, Western blot analysis showed that CA-induced phosphorylation of IRF-3 was suppressed following STING knockdown (Figure 6B), and densitometric quantification further confirmed this reduction (Figure 6C). To investigate downstream consequences of STING activation, we examined the transcription of NOS genes and NO production. qRT-PCR results showed that CA treatment upregulated the mRNA levels of nNOS, eNOS, and iNOS, while STING knockdown reversed this upregulation (Figure 6D,E). In parallel, Griess assay confirmed that CA-induced NO generation was significantly diminished in STING-silenced cells (Figure 6F,G). We further assessed whether STING knockdown affects CA-induced apoptosis. Western blot analysis demonstrated that CA treatment increased the expression of cleaved PARP, cleaved caspase-3, Bax, and Bak, and decreased Bcl-2 and Bcl-xL levels, whereas these alterations were partially reversed in the siSTING + CA group (Figure 6H). Densitometric analysis confirmed the rescue effects of STING silencing on apoptosis-related protein expression (Figure 6I). Moreover, flow cytometry analysis revealed that STING knockdown significantly reduced the proportion of total apoptotic cells (early + late apoptosis) induced by CA in both HOS and MG63 cells (Figure 6J,K). Collectively, these findings indicate that CA-induced NOS expression, NO production, and mitochondrial apoptosis are largely dependent on STING/IRF-3 signaling, suggesting that STING plays an essential role in mediating the pro-apoptotic effects of CA in osteosarcoma cells.

### 3.7. CA Inhibits Osteosarcoma Growth In Vivo via Activation of the STING/IRF-3 Signaling Pathway

**Figure 7:**
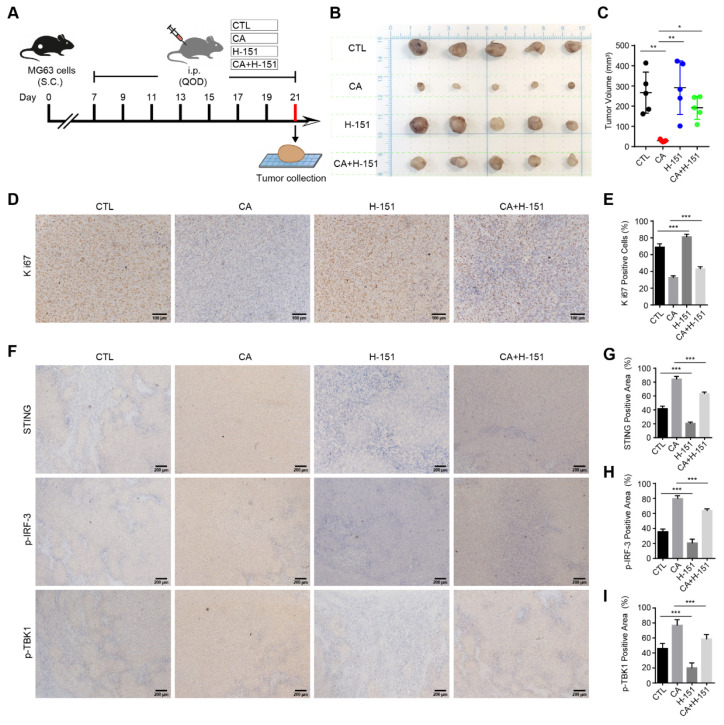
CA inhibits tumor growth in vivo via activation of the STING signaling pathway. (**A**) Schematic of in vivo study design. (**B**) Gross tumor images from each treatment group at the experimental endpoint. (**C**) Statistical analysis of tumor volume at the experimental endpoint. Data are presented as scatter plots with mean ± SD (*n* = 5). (**D**) Representative IHC images of Ki67 in tumor tissues (scale bar = 100 μm). (**E**) Quantification of Ki67-positive cells. (**F**) IHC staining of STING, p-TBK1, and p-IRF-3 (scale bar = 200 μm). (**G**–**I**) Quantitative analysis of IHC staining areas for STING, p-IRF-3, and p-TBK1. Data are presented as mean ± SD (*n* = 5). * *p* < 0.05, ** *p* < 0.01, *** *p* < 0.001 versus PBS group.

To assess the in vivo antitumor activity of CA and validate the involvement of the STING pathway, a subcutaneous xenograft model was established by injecting MG63 osteosarcoma cells into BALB/c nude mice. Once tumors formed, mice were randomly divided into four groups (*n* = 5 per group) and treated intraperitoneally every other day for 2 weeks with PBS (vehicle), CA (30 mg/kg), H-151 (10 mg/kg), or a combination of CA and H-151 (Figure 7A). As shown in Figure 7B,C, CA markedly suppressed tumor growth compared with the PBS group (*p* < 0.01), as evidenced by significantly reduced tumor volumes at the study endpoint. Notably, co-administration of the STING inhibitor H-151 partially abrogated the antitumor effect of CA, suggesting that CA’s efficacy is, at least in part, mediated through STING activation. IHC staining revealed a pronounced reduction in Ki67 expression, a proliferation marker, in the CA-treated group, which was partially restored upon H-151 co-treatment (Figure 7D,E). Furthermore, IHC analysis showed that CA significantly upregulated STING, p-TBK1, and p-IRF3 expression within tumor tissues, whereas these increases were markedly suppressed by H-151 co-treatment (Figure 7F–I). Collectively, these findings indicate that CA exerts potent antitumor effects in vivo through activation of the STING/IRF3 signaling pathway, and that pharmacological inhibition of STING by H-151 attenuates this therapeutic benefit.

## 4. Discussion

Osteosarcoma is an aggressive primary bone tumor for which treatment advances have been limited in recent decades [31]. The search for new therapeutic agents that can both suppress tumor progression and modulate the tumor immune microenvironment remains a priority for improving clinical outcomes. In the present work, we identified for the first time that CA, a phenolic diterpenoid compound derived from *Rosmarinus officinalis*, exerts potent anti-osteosarcoma activity through a concerted mechanism involving activation of the STING/IRF-3 pathway, induction of NO production, and mitochondrial-mediated apoptosis. Notably, recent findings have suggested that CA directly targets the C-terminal tail of STING and promotes its activation, providing strong mechanistic support for our observations [24]. Although that study primarily focused on inflammatory disease models, the demonstration of CA as a direct STING agonist strengthens the plausibility of our proposed pathway in osteosarcoma cells.

Previous investigations have documented the anti-proliferative and pro-apoptotic activities of CA in a variety of malignancies, such as lung, colon, gastric, and melanoma cancers [32,33]. Our research extends these findings to osteosarcoma and highlights a previously unrecognized mechanism by which CA exerts its antitumor effects—through direct engagement of the STING pathway. The STING axis plays a critical role in innate immunity and cancer immunosurveillance [34]. Upon activation, STING interacts with and activates TBK1, which subsequently phosphorylates IRF-3, promoting its nuclear translocation and initiating transcription of type I interferons and other pro-inflammatory mediators [35]. Recent studies have explored pharmacological STING agonists as promising cancer immunotherapeutic agents [9,11,35]. In our study, CA significantly increased STING, TBK1, and phosphorylated IRF-3 protein levels in osteosarcoma cells, and this activation was abrogated by the STING inhibitor H-151, affirming that CA functions, at least in part, as a STING activator. While CA’s anticancer potential has been widely studied in other tumor types, its effect on osteosarcoma remains largely unexplored. A prior study reported that CA selectively reduced the viability of osteosarcoma cells in both 2D and 3D culture systems, while exhibiting less cytotoxicity toward healthy bone-derived cells, suggesting differential tolerance and potential tumor selectivity [25]. Our findings build upon this early evidence and provide mechanistic and in vivo validation that expand CA’s anticancer spectrum into the osteosarcoma context.

Importantly, we discovered that STING activation by CA led to robust upregulation of inducible iNOS and increased production of NO. Nitric oxide plays a dual role in cancer; low levels support angiogenesis and tumor survival, while high levels promote DNA damage, oxidative stress, and apoptosis [13,14,15]. Our data indicate that CA induces high-level NO production sufficient to trigger apoptosis in osteosarcoma cells, which was mitigated by L-NMMA, an NOS inhibitor. These findings align with previous reports that STING signaling can transcriptionally upregulate iNOS expression through IRF-3 and NF-κB-dependent mechanisms [12,36]. Beyond direct induction of tumor cell death, STING pathway activation has also been shown to remodel the tumor microenvironment [37]. Recent work demonstrated that STING-activating nanoparticles not only enhanced nitric oxide–driven tumor clearance but also normalized the vascular-immune interface, thereby amplifying the efficacy of immune checkpoint inhibitors such as anti–PD-1/PD-L1 therapy [38]. This underscores the potential of STING-targeted interventions, including CA, to elicit both cytotoxic and immunomodulatory antitumor responses.

In the present study, we demonstrate that CA induces pronounced apoptosis in osteosarcoma cells through activation of the STING/TBK1/IRF3 signaling pathway, accompanied by a significant increase in intracellular NO levels. Pharmacological inhibition or siRNA-mediated knockdown of STING markedly attenuated CA-induced NO production and apoptotic cell death, indicating that NO acts as a critical downstream effector of STING activation in this context. Notably, our results showed that CA treatment upregulated the expression of all three NOS isoforms, including nNOS, eNOS, and iNOS. However, among these isoforms, iNOS exhibited the most prominent induction following CA exposure. This observation is of particular relevance, as iNOS is well recognized as the primary source of sustained and high-output NO production in tumor cells, in contrast to the low-level and tightly regulated NO generated by nNOS and eNOS under physiological conditions. In cancer cells, iNOS-derived NO has been widely implicated in the regulation of redox stress and apoptosis [39,40]. Therefore, although CA broadly enhances NOS expression, our data support the notion that iNOS is the dominant contributor to the elevated NO levels observed in CA-treated osteosarcoma cells. Consistent with this interpretation, pharmacological blockade of NO synthesis using the NOS inhibitor L-NMMA significantly reversed CA-induced mitochondrial membrane potential collapse, cytochrome c release, and caspase-dependent apoptosis, further confirming a central role for NO in mediating CA-triggered mitochondrial apoptotic signaling. Together, these findings establish a mechanistic link between STING activation and iNOS-dependent NO overproduction, which ultimately drives mitochondrial apoptosis in osteosarcoma cells.

Beyond STING/IRF3 signaling, recent studies have demonstrated that CA modulates additional redox-sensitive pathways, particularly the PTEN/AKT axis [41]. For example, CA has been shown to upregulate PTEN phosphorylation and suppress AKT activation in bone-related pathological models, primarily through attenuation of ROS signaling. Interestingly, in that context, CA exhibited minimal effects on canonical NF-κB activation, suggesting that its regulatory effects may preferentially target redox-sensitive survival pathways rather than broadly suppress inflammatory transcription factors. Given that ROS serves as a common upstream modulator of both STING activation and PI3K/AKT signaling, it is conceivable that CA-mediated redox modulation may coordinate innate immune activation with suppression of pro-survival AKT signaling in osteosarcoma cells. Although MAPK pathways such as JNK and p38 have been implicated in bone tumor progression, their interaction with STING signaling in osteosarcoma remains to be clarified. Further studies integrating phospho-proteomic or pathway inhibition approaches will be necessary to delineate the relative contribution of these signaling networks.

**Figure 8:**
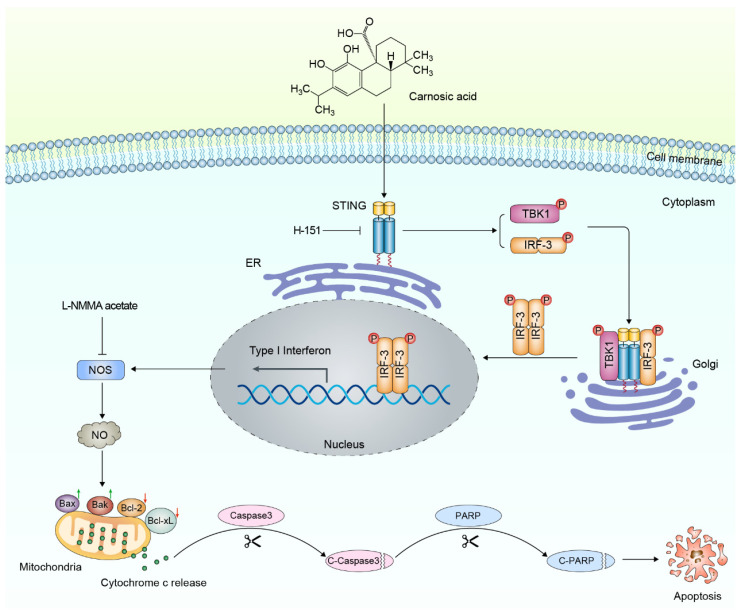
Proposed mechanism by which CA induces apoptosis in osteosarcoma cells via the STING/IRF-3 signaling axis and NO production. CA activates the STING pathway, leading to phosphorylation of TBK1 and IRF-3, nuclear translocation of p-IRF-3, and upregulation of type I interferons and proinflammatory cytokines. Simultaneously, STING activation promotes iNOS expression and enhances NO production. Elevated NO induces mitochondrial outer membrane permeabilization (MOMP), resulting in cytochrome c release, caspase-3 cleavage, and subsequent PARP cleavage, culminating in apoptosis. This cascade is attenuated by the STING inhibitor H-151 and the NOS inhibitor L-NMMA. Additionally, CA modulates the expression of Bcl-2 family proteins by increasing pro-apoptotic Bax and Bak while decreasing anti-apoptotic Bcl-2 and Bcl-xL, further contributing to mitochondrial apoptosis. Green upward arrows indicate upregulation of molecular expression or activity, whereas red downward arrows indicate downregulation.

Mechanistically, CA-induced NO appears to promote mitochondrial outer membrane permeabilization (MOMP), enabling cytochrome c release and subsequent activation of caspase-3, culminating in PARP cleavage—key events in mitochondrial apoptosis. This process was accompanied by increased expression of pro-apoptotic proteins (Bax, Bak) and suppression of anti-apoptotic factors (Bcl-2, Bcl-xL), corroborating the engagement of the intrinsic apoptotic pathway (Figure 8). The elevated levels of cleaved caspase-3 and cleaved PARP in CA-treated cells further validated the involvement of the mitochondrial apoptotic cascade. In addition to its tumoricidal effects, STING activation may also enhance antitumor immunity. Previous studies have shown that STING signaling in tumor and stromal cells stimulates type I IFN production, T cell infiltration, and tumor regression [11,42]. Notably, potent STING activation has also been linked to the induction of immunogenic cell death (ICD), characterized by the release of damage-associated molecular patterns (DAMPs) that promote dendritic cell maturation and adaptive immune priming. For instance, Wang-Bishop et al. demonstrated in a neuroblastoma model that strong STING activation led to robust ICD and enhanced antitumor immune responses [43]. These findings support our hypothesis that CA, through STING/IRF3/NO-mediated apoptosis, may also trigger ICD and thereby contribute to a more immunostimulatory tumor microenvironment.

Recent single-cell RNA sequencing analyses have revealed significant cellular heterogeneity within osteosarcoma tissues, identifying multiple malignant and non-malignant cell populations, including mesenchymal progenitors, immune infiltrates, endothelial cells, and tumor-associated stromal cells [44,45]. Although our study primarily investigated tumor-intrinsic STING signaling in osteosarcoma cell lines, it is plausible that CA may exert broader effects within the tumor microenvironment. Given the established role of STING in innate immune activation, CA-mediated STING activation could potentially modulate immune cell subsets, cytokine production, or stromal remodeling. Future studies employing single-cell approaches or immunocompetent models will be necessary to dissect these complex interactions.

While this study provides important mechanistic insights, several limitations should be acknowledged. First, although our data strongly suggest STING pathway activation by CA, direct biochemical evidence of CA–STING binding remains to be established through molecular docking or structural studies, such as surface plasmon resonance or co-crystallization with the STING protein. Moreover, although STING involvement was validated using siRNA-mediated knockdown and pharmacologic inhibition with H-151, additional genetic approaches such as multiple independent siRNAs, rescue experiments with siRNA-resistant constructs, or CRISPR-mediated knockout would further strengthen causal inference. Second, while CA-induced NOS transcription and NO production were significantly attenuated by STING knockdown, direct transcriptional regulation of NOS genes by IRF3 was not experimentally confirmed. Chromatin immunoprecipitation or promoter-reporter assays would be required to determine whether IRF3 directly binds to NOS promoters. In addition, although L-NMMA was used to demonstrate functional dependence on NOS activity, it does not distinguish among individual NOS isoforms. Isoform-specific genetic silencing or selective inhibitors would help clarify whether iNOS, eNOS, or nNOS serves as the dominant mediator of apoptosis in this context. Third, the upstream trigger responsible for STING activation by CA remains undefined. Future studies should investigate whether CA induces cytosolic DNA accumulation, modulates cGAS activity, or enhances cGAMP production to achieve mechanistic completeness. Finally, the in vitro nature of our cellular experiments and the use of immunodeficient nude mice limit the ability to assess contributions from adaptive immune components such as T cells or dendritic cells. Future investigations employing immunocompetent or syngeneic animal models will be essential to evaluate the immunomodulatory impact of CA within a physiologically relevant tumor microenvironment.

## 5. Conclusions

Taken together, our findings demonstrate that CA exerts potent antitumor effects in osteosarcoma through a coordinated cascade involving STING/IRF-3 activation, increased NO production, mitochondrial-mediated apoptosis, and the possible induction of immunogenic cell death. These data highlight CA as a promising candidate for STING-targeted therapy, with potential value both as a monotherapy and in rational combinations with existing immunotherapies. Notably, recent reviews have emphasized the clinical momentum of STING agonists as adjuvants to immune checkpoint blockade, citing their ability to enhance type I interferon production, promote dendritic cell maturation, and improve T cell infiltration [23]. This supports the rationale for future investigations into combining CA with PD-1/PD-L1 inhibitors in osteosarcoma and other STING-responsive malignancies. Moving forward, rigorous in vivo validation and translational studies will be critical to advancing CA toward clinical development as part of next-generation immuno-oncology strategies.

## Data Availability

The original contributions presented in this study are included in the article. Further inquiries can be directed to the corresponding author.

## Supplementary Materials

The following supporting information can be downloaded at: https://www.mdpi.com/article/10.3390/antiox15030374/s1, Figure S1: Screening of four independent siRNAs targeting STING in osteosarcoma cells. (A) Western blot analysis of STING protein expression in HOS cells transfected with four candidate siRNAs targeting human STING (STING-homo-679, STING-homo-806, STING-homo-886, and STING-homo-1047). (B) Densitometric quantification of STING protein levels in HOS cells normalized to α-Tubulin. (C) Western blot analysis of STING expression in MG63 cells following transfection with the same four siRNA candidates. (D) Densitometric quantification of STING protein levels in MG63 cells normalized to α-Tubulin.

## Abbreviations

The following abbreviations are used in this manuscript:

OS	Osteosarcoma
CA	Carnosic acid
hBMSCs	Human bone marrow-derived mesenchymal stem cells
NO	Nitric oxide
iNOS	Inducible nitric oxide synthase
STING	Stimulator of interferon genes
TBK1	TANK-binding kinase 1
IRF3	Interferon regulatory factor 3
IFN-I	Type I interferon
DMSO	Dimethyl sulfoxide
DMEM	Dulbecco’s Modified Eagle Medium
FBS	Fetal bovine serum
PBS	Phosphate-buffered saline
siRNA	Small interfering RNA
qRT-PCR	Quantitative real-time polymerase chain reaction
BSA	Bovine serum albumin
H&E	Hematoxylin and eosin
DAB	3,3′-diaminobenzidine
HRP	Horseradish peroxidase
GAPDH	Glyceraldehyde 3-phosphate dehydrogenase
ROS	Reactive oxygen species
