# Supplementary material for: Carnosic Acid Activates the STING/IRF3 Pathway to Induce Nitric Oxide-Mediated Apoptosis in Osteosarcoma Cells
Source: Antioxidants (Basel). 2026 Mar 16;15(3):374. doi: 10.3390/antiox15030374 (PMC13024143; doi:10.3390/antiox15030374)
Supplement: Supplementary file 1 [file antioxidants-15-00374-s001.zip › antioxidants-4143218-supplementary.pdf]

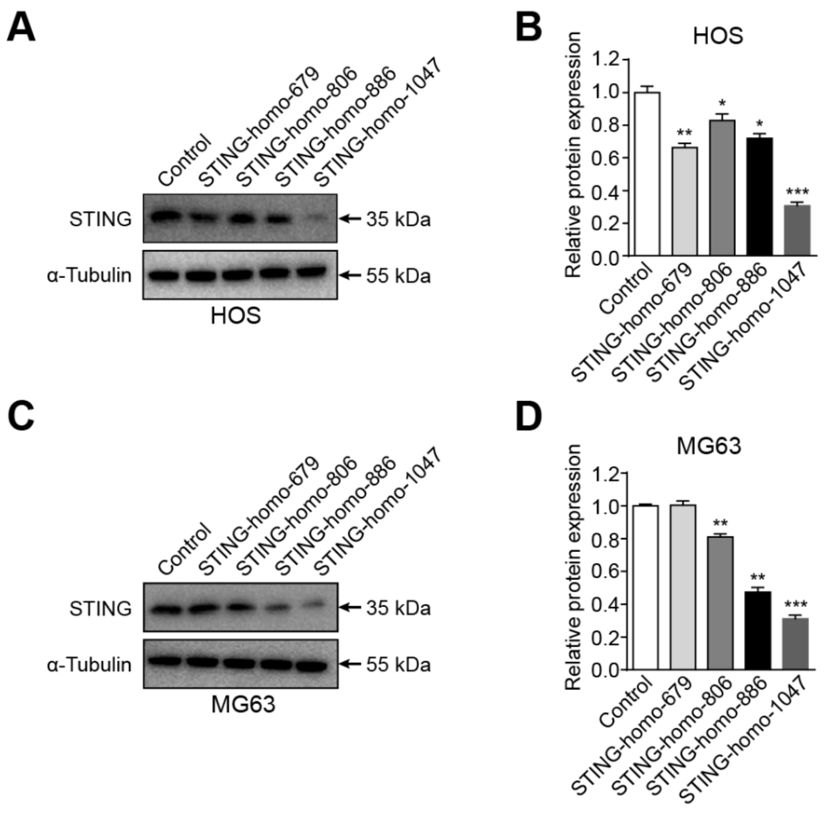

Figure S1. Screening of four independent siRNAs targeting STING in osteosarcoma cells. (A) Western blot analysis showing STING protein expression in HOS cells transfected with four candidate siRNAs targeting human STING (STING-homo-679, STING-homo-806, STING-homo-886, and STING-homo-1047). α-Tubulin was used as the loading control. (B) Densitometric quantification of STING protein levels in HOS cells shown in panel (A). Band intensities were quantified using ImageJ and normalized to α-Tubulin. (C) Western blot analysis of STING expression in MG63 cells following transfection with the same four siRNA candidates. α-Tubulin served as the loading control. (D) Densitometric quantification of STING protein levels in MG63 cells shown in panel (C). Among the four tested sequences, STING-homo-1047 exhibited the highest knockdown efficiency in both HOS and MG63 cells and was therefore selected for subsequent mechanistic experiments. Data are presented as mean ± SD from three independent biological experiments (n = 3). Statistical significance was determined by one-way ANOVA followed by Dunnett's post hoc test versus the control group. * $p < 0.05$, ** $p < 0.01$, *** $p < 0.001$.
